# Atomically Resolved Electron Reflectivity at a Metal/Semiconductor Interface

**DOI:** 10.1002/advs.202515182

**Published:** 2025-11-29

**Authors:** Ding‐Ming Huang, Jian‐Huan Wang, Jie‐Yin Zhang, Yuan Yao, Hongqi Xu, Jian‐Jun Zhang

**Affiliations:** ^1^ Beijing Academy of Quantum Information Sciences Beijing 100193 China; ^2^ Beijing National Laboratory for Condensed Matter Physics and Institute of Physics Chinese Academy of Sciences Beijing 100190 China; ^3^ Beijing Key Laboratory of Quantum Devices and School of Electronics Peking University Beijing 100871 China; ^4^ Hefei National Laboratory Hefei 230088 China

**Keywords:** metal/semiconductor interface, interfacial characterization, scanning tunneling microscopy, coherent tunneling, quantum well, interfacial scattering

## Abstract

An atomically flat interface is achieved between face‐centered cubic Al and diamond lattice Ge via molecular beam epitaxy (MBE). Based on the measurements of scanning tunneling microscopy (STM), an atomically resolved lateral periodic change of the electron reflectivity at the Al/Ge interface is demonstrated. The relative variation of electron reflectivity is up to ≈22% in a lateral 2 nm. It is speculated that the change of reflectivity results from the local electronic states at the Al/Ge interface. This phenomenon provides an atomically non‐destructive method for detecting the buried interfacial states in hetero‐structures by STM.

## Graphical abstract

An atomically flat interface is achieved in the incoherent Al/Ge heterostructure using molecular beam epitaxy (MBE). Scanning tunneling microscopy (STM) reveals a laterally periodic modulation of electron reflectivity with atomic resolution at the commensurate Al/Ge interfacial lattice, originating from the local electronic states. This phenomenon provides a method for detecting buried interfacial states in heterostructures.

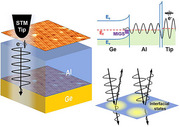

## 1 Introduction

Epitaxial metal thin films on semiconductor substrates exhibit exotic physical properties.^[^ ^1^, ^2^, ^3^, ^4^, ^5^, ^6^ ^]^ Due to vertical thickness quantum confinement and lateral lattice modulations by substrates, the electronic properties of these metal thin films are strongly related to the crystalline quality and lattice structures at the interfaces. Epitaxially grown Pb and Al thin films on Si substrate are two well‐studied examples, where quantum size effect induced modulations of electron‐phonon coupling (EPC)^[^ ^1^, ^2^ ^]^ and superconductivity,^[^ ^3^, ^4^ ^]^ the emergence of type‐II superconductivity,^[^ ^4^, ^5^ ^]^ and Mott transition^[^ ^6^ ^]^ were experimentally observed. Superconductor/Ge‐Si heterostructures have also been utilized in constructing advanced quantum devices, such as Josephson field‐effect transistors,^[^ ^7^ ^]^ superconducting diodes,^[^ ^8^ ^]^ and superconducting transmon qubits,^[^ ^9^ ^]^ where high‐quality materials are essential for achieving high performance. Non‐destructive characterizations of interfacial structures and properties in these materials are essential. Over the past two decades, studies have demonstrated that the interfacial lattice structures between Si substrate and epitaxial metal (Pb, In, Cd, and Al) thin films can be observed through scanning tunneling microscopy (STM) surface imaging,^[^ ^10^, ^11^, ^12^, ^13^, ^14^, ^15^, ^16^, ^17^ ^]^ even if the films are up to a few dozen atomic layers thick.^[^ ^10^ ^]^ The STM visualizing of the buried interfaces relies on two types of mechanisms. First, in the condition of a metal film grown on a reconstructed substrate, the visualization of the buried interface results from both lateral change of film thickness^[^ ^10^, ^12^, ^13^ ^]^ and local electron diffuse scattering at the interfacial vacancies, where the diffuse scattering may also be related to local electron‐phonon scattering (EPS).^[^ ^14^ ^]^ Such interfaces typically lead to position‐dependent energy shifts of quantum well (QW) peaks in the differential conductance (dI/dV) spectrum or intensity modulations in the second derivative d^2^I/dV^2^ spectrum. Second, in the condition of an adiabatic interface,^[^ ^15^ ^]^ where interfacial vacancies are absent, the interface visualization is achieved via measuring the lateral phase shift in the electron reflective scattering, which is sensitively modulated by the interfacial atomic arrangements.^[^ ^15^, ^16^, ^17^ ^]^ This type of interface also induces position‐dependent peak shifts in the dI/dV spectrum.

Here, we report the epitaxial growth of single‐crystalline Al(111) films on a Ge(111) substrate, as well as an experimental study of the interfacial properties of the as‐grown samples by STM and scanning tunneling spectroscopy (STS) measurements. A distinct Moiré pattern of the interfacial Al/Ge lattice is observed on the Al surface by STM, which is persistent for film thicknesses ranging from 1 to 25 nm (4–100 monolayers). Based on the STM and STS measurements, we demonstrate spatial uniformity in both the dI/dV peak positions and the d^2^I/dV^2^ intensity, indicating the presence of an adiabatic and reflective‐phase‐homogeneous Al/Ge interface. Thus, the observed Moiré pattern in our system is independent of local electron diffuse scattering and reflective phase shift induced by interfacial atomic arrangements, in contrast to previous studies. By considering the Al film as a Fabry‐Perot interferometer, we have introduced a model to describe the electron transmission in the STM measurements and shown that such a model qualitatively reproduces our experimental observations. These results allow us to attribute the Moiré pattern to the atomically resolved lateral changes of electron reflectivity at the perfect Al/Ge interfaces.

## 2 Results and Discussion

**Figure 1:**
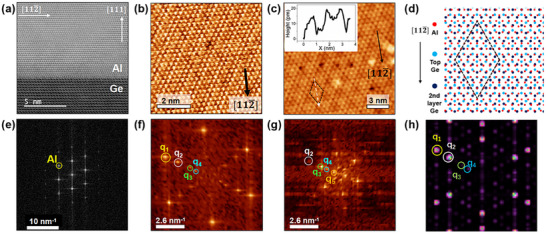
Epitaxial Al film on Ge(111). a) Atomic resolution cross‐sectional TEM image of the Al/Ge interface. b) Atomic resolution STM image of a 2 nm thick Al/Ge film (taken at sample bias of *V*_s_ = −70 mV and tunneling current of *I*_t_ = −1 nA). c) STM topographic image of the 2 nm Al/Ge film (*V*_s_ = −60 mV, *I*_t_= −318 pA). A 2 nm periodic supercell of the surface pattern is marked by a dashed diamond. Inset: height profile along the white dashed arrow. d) Schematic of the in‐plane atomic arrangement at the Al/Ge interface. The gray dashed diamond marks a 2 nm supercell. e) FFT image of the Al lattice from (a), showing only the Al reciprocal lattice points. f–h) FFT images of the structures in (b–d), respectively, indicating the influence of the Ge substrate on the STM contrast. The reciprocal lattice points of Al and Ge are denoted as q_1_ and q_2_, respectively. q_3_, q_4_ and q_5_ are derived points of q_3_ = 2q_2_‐q_1_, q_4_ = q_1_‐q_2_ and q_5_ = q_3_‐q_4_, respectively.

To obtain a single‐crystalline Al film on Ge, a 30 nm thick Ge buffer is first grown on Ge(111) using molecular beam epitaxy (MBE) to achieve a high‐quality Ge surface. The sample is then in situ transferred to another MBE chamber for Al deposition. The Al film is deposited at 40 °C with a growth rate of 1.0 Å s^−1^. The thickness of the Al layers is determined by the deposition time. **Figure** **1**a shows a cross‐sectional transmission electron microscope (TEM) image of the Al/Ge interface. It is seen that the face‐centered cubic (FCC) Al perfectly sits on the diamond‐structured Ge with an atomically sharp interface. STM measurements are performed in an in situ chamber at a temperature of 10 K. Figure 1b shows the STM image of a 2 nm thick Al film obtained with a Ge‐atom‐decorated tip, offering atomic resolution. The close‐packed lattice of Al(111) with periodic modulations is resolved. Figure 1c shows the large scale STM topography of the same sample, obtained with an Au‐coated tip. Here, a 2 nm periodic pattern is visible, and the black dashed‐line diamond marks a unit cell. The height profile across the unit cell is shown in the inset of Figure 1c. Figure 1f shows the corresponding fast Fourier transforms (FFT) of Figure 1b. The reciprocal lattice points with the highest intensity (q_1_) correspond to the Al(111) lattice. Three sets of reciprocal points are observed within the Brillouin zone, and q_2_ matches the reciprocal points of the bulk Ge(111) lattice. The q_3_ and q_4_ points correspond to the reciprocal points of the Moiré period, with q_3_ = 2q_2_‐q_1_ and q_4_ = q_1_‐q_2_. These reciprocal points indicate that the buried Ge substrate has a significant impact on the STM measurements. For comparison, Figure 1e shows the FFT of the Al lattice from Figure 1a, revealing only the periodicity of the Al lattice. Temperature‐dependent low‐energy electron diffraction (LEED) and STM measurements further confirm a single‐crystalline Al lattice without additional periodic structures, as detailed in Section S3 (Supporting Information). The TEM and LEED measurements indicate negligible structural modulation in the Al lattice. Therefore, the superstructure observed in STM images is related to the electronic states. Figure 1g shows the FFT of a defect‐free region in Figure 1c, obtained from an area at the same scale as that in Figure 1b. In Figure 1g, the Moiré period of q_5_ = q_3_‐q_4_ is observed, but the q_1_ points disappear. This is caused by the reduced spatial resolution of the Au‐coated tip. The enhanced intensities of the q_3_ and q_5_ points correspond to the 0.68 and 2 nm periodicities in Figure 1c. Figure 1d schematically shows the possible atomic arrangement (top‐view) at the Al/Ge interface with a unit cell marked by a dashed diamond. The lattice constants on (111) facets of bulk Al and Ge are 2.86 and 4.00 Å, respectively. A 7‐Al‐atom lattice on Al(111) matches to a 5‐Ge‐atom lattice on Ge(111) (mismatch is only ≈0.1%), forming a commensurate 2 nm periodicity, as confirmed by the TEM (Figure 1a). A simulation of the Moiré pattern based on this lattice configuration is provided in Section S1 (Supporting Information). Figure 1h shows the corresponding FFT of Figure 1d. The highest‐intensity reciprocal points within the Ge Brillouin zone match the q_3_ and q_4_ points in the measurements. Temperature‐dependent STM studies demonstrate that the surface pattern correlates with vertically coherent electronic states in the material, confirming the influence of the Al/Ge interface on the STM results, with details presented in Section S2 (Supporting Information). Therefore, the STM pattern is attributed to the visualization of the Al/Ge interface.

**Figure 2:**
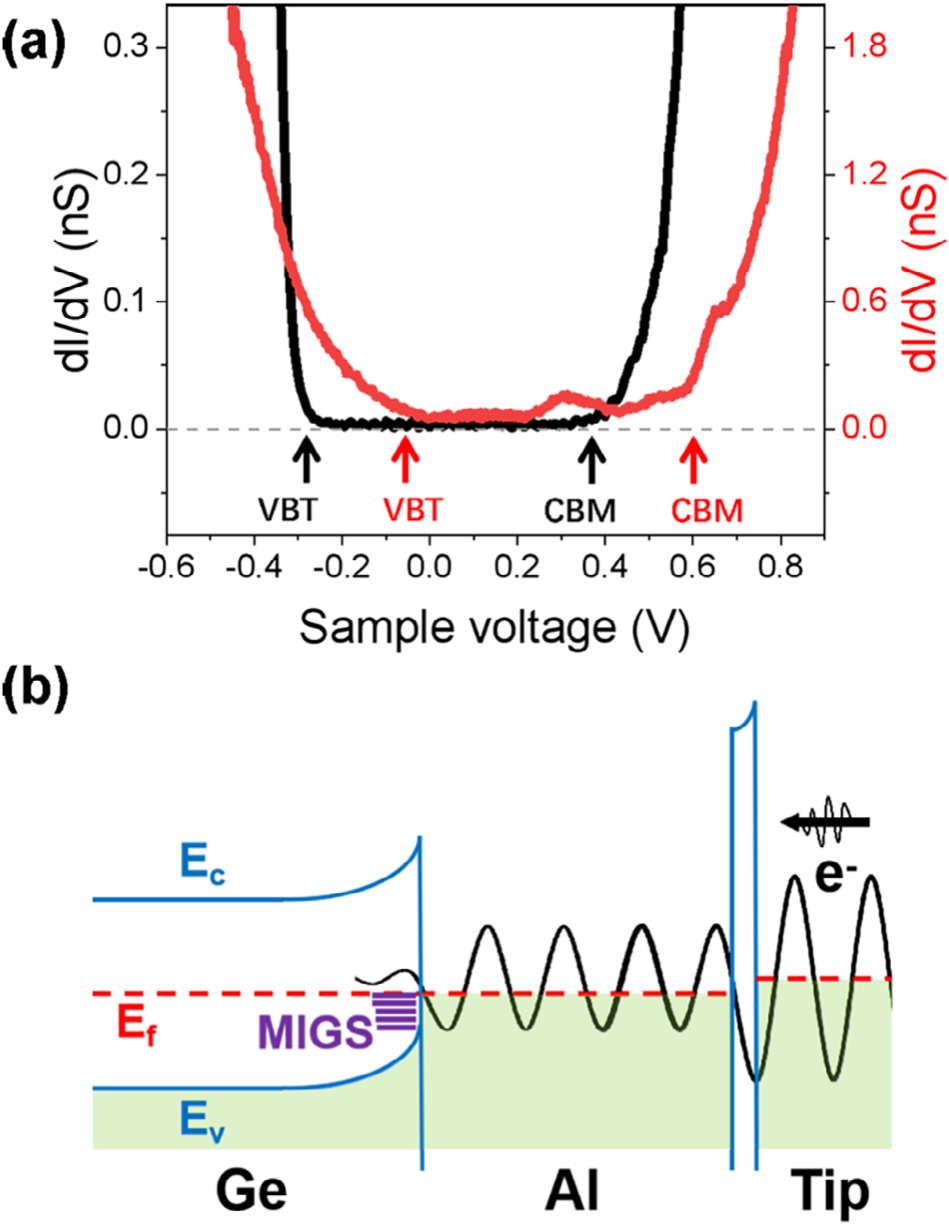
Al/Ge Interfacial states. a) Evidence of Fermi‐level pinning at the Al/Ge interface. The dI/dV spectra of the intrinsic Ge(111) surface (*V*_s_ = 570 mV, *I*_t_ = 135 pA, and bias modulation of *V*_mod_ = 30 mV) and the 0.8‐monolayer‐Al‐covered Ge (*V*_s_ = 970 mV, *I*_t_ = 405 pA, and *V*_mod_ = 5 mV) are shown in black and red curves, respectively. The VBT and conduction‐band minimum (CBM) are marked by arrows. b) Schematic band diagram of an Al/Ge film and the transmission process of a coherent electron in STM measurements. A coherent electron can experience multiple reflections in the Al film, and the transmissivity from the tip to the MIGS depends on the interfacial reflectivity.

The variation in the Ge band edge with Al deposition is characterized by STS. As shown in **Figure** **2**a, the black curve corresponds to the dI/dV spectrum taken from an intrinsic epitaxial Ge film, while the red curve represents the spectrum from Ge covered with 0.8 monolayer of Al. The voltage range with zero dI/dV values in the black curve corresponds to the energy bandgap of Ge, showing a valence band top (VBT) at −0.28 eV. The red curve exhibits significantly low dI/dV values within the bias range of −0.06 to +0.59 V, beyond which the values increase rapidly by approximately an order of magnitude. We speculate that the turning points in the red curve indicate the band edges of the underlying Ge, and the increased dI/dV beyond these points is attributed to the enhanced tunneling from the semiconductor Ge. Based on this interpretation, the VBT shifts by 0.22 eV toward the Fermi‐level with Al coverage. The measured VBT value is nearly identical to that reported for Fermi‐level‐pinned metal/Ge interfaces,^[^ ^18^, ^19^ ^]^ serving as strong evidence of Fermi‐level pinning at this commensurate interface. Figure 2b schematically shows the band diagram. Metal‐induced gap states (MIGS) form on the Ge side with a penetration depth of several atomic layers,^[^ ^18^, ^20^, ^21^ ^]^ inducing band bending. The MIGS result in modulations in the electron transmission at the interface, which have a significant impact on the coherent electron tunneling in the Al film.

**Figure 3:**
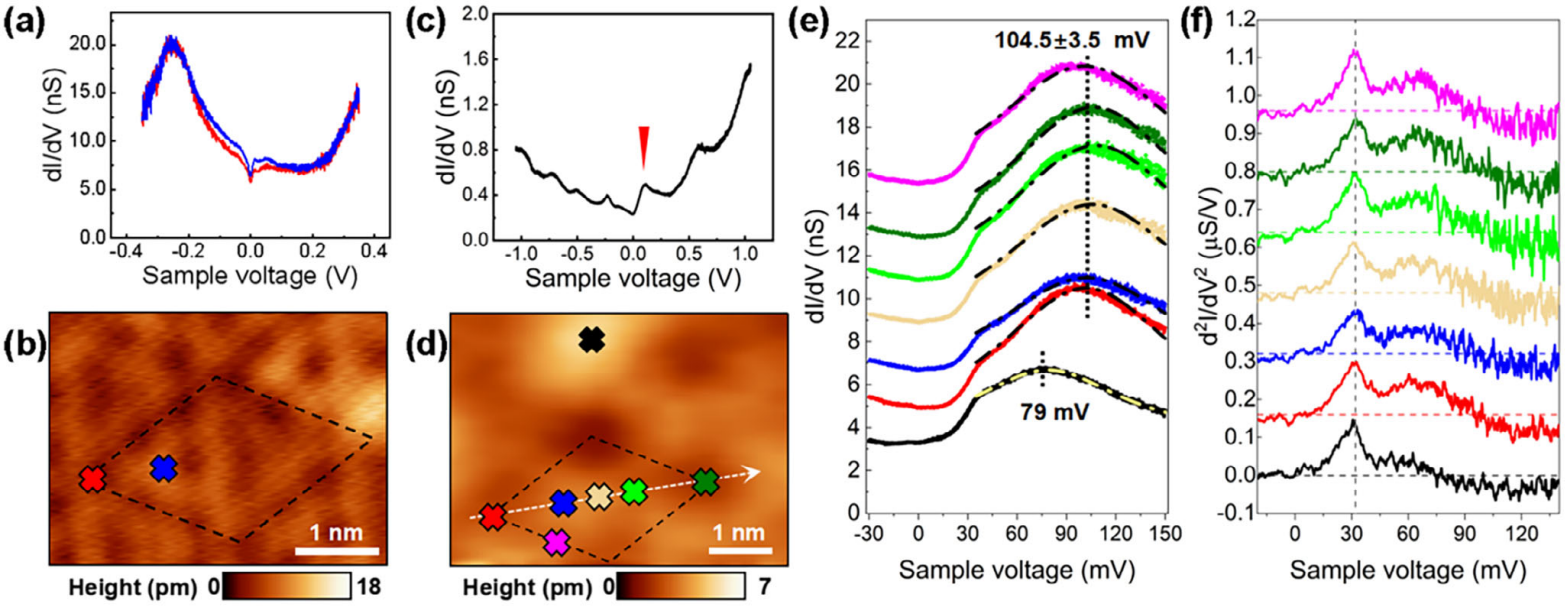
Local STS spectra on the surface pattern. a) Local dI/dV spectra of a 2 nm Al/Ge film (stabilized at *V*_s_ = −50 mV, *I*_t_ = −436 pA, bias modulation of *V*_mod_ = 6 mV). The tip height was kept constant during these measurements. The peak width of the QW state changes with lateral position. b) STM image (*V*_s_ = −30 mV, *I*_t_ = −246 pA) of the corresponding measurement positions for the spectra in (a). c) dI/dV spectrum on a 10 nm thick Al film (*V*_s_ = −450 mV, *I*_t_ = −125 pA and *V*_mod_ = 25 mV). The oscillation peaks arise from tunneling through QW states. The red triangle marks the QW peak studied in (e). d) STM image of a 2 nm periodic supercell of the surface pattern acquired with a blunt tip (*V*_s_ = −50 mV, *I*_t_ = −65 pA). Colored crosses mark the positions for STS measurements. e) Local dI/dV spectra (*V*_s_ = −50 mV, *I*_t_ = −165 pA, *V*_mod_ = 5 mV) acquired at positions marked in (d), shown in corresponding colors. These curves are vertically shifted by 2 nS for clarity. The dot‐dashed curves are fits to the data. The dI/dV peaks obtained in the supercell exhibit the same peak position of 104.5 ± 3.5 mV, whereas a peak shift of −25.5 mV occurs at the position containing a Ge dopant (black curve). f) Corresponding d^2^I/dV^2^ spectra obtained by numerical differentiation of the dI/dV spectra in (e). These curves are vertically shifted by 0.16 µS V^−1^ for clarity. The peak at 32 mV is attributed to EPS in the Al film. The peak values are independent of the lateral position.

STS measurements are performed to study the electronic states, using a standard lock‐in technique. The dI/dV spectra show film‐thickness‐dependent peaks, revealing the QW states in Al films, with details presented in Section S8 (Supporting Information). **Figure** **3**a,b shows the local dI/dV spectra obtained on the 2 nm Al/Ge sample and the corresponding measuring positions (marked with the same color), respectively. The tip height was kept constant during the measurements presented in Figure 3a. These dI/dV spectra exhibit a QW peak at −0.25 V. The peak width changes with measuring position, while the peak position remains unchanged. This results in higher dI/dV values near the Fermi‐level at the position with higher apparent height (blue site). A zero‐bias conductance dip (ZBCD) is observed in these dI/dV curves, which is commonly observed in various metal films.^[^ ^22^, ^23^ ^]^

To study coherent electronic states with energies comparable to those of the MIGS, STS measurements are performed on a 10 nm thick Al/Ge sample, where a QW peak lies ≈100 mV above the Fermi‐level as marked by the red triangle in Figure 3c. A pre‐treated blunt tip is used to study the local STS spectra, ensuring high energy precision, with details presented in Section S4 (Supporting Information). The reduced spatial resolution suppresses the influence of the surface Al lattice on the position‐dependent differential conductance,^[^ ^13^ ^]^ enhancing the contribution from QW states to the dI/dV results. Figure 3e shows a set of dI/dV spectra obtained from different positions within a Moiré pattern supercell, corresponding to the colored cross markers in Figure 3d. To minimize measurement error, the tip height is readjusted by the feedback current before each individual measurement in Figure 3e. A detailed discussion regarding the influence from current feedback is provided in Section S8 (Supporting Information). The dI/dV peaks at different positions appear almost at the same sample bias of 104.5 ± 3.5 mV, with error analysis presented in Section S5 (Supporting Information). If the ± 3.5 mV deviation is attributed to a reflective phase shift of ≈2*π*/100, this deviation remains significantly smaller than those reported in comparable material systems,^[^ ^15^, ^16^ ^]^ demonstrating an atomically flat Al/Ge interface with negligible lateral variation of the reflective phase. As a comparison, the dI/dV spectrum (black curve in Figure 3e) taken at an atomic disorder site, most probably a Ge dopant or an interfacial adatom (see Section S6, Supporting Information), shows a large QW energy shift of −25.5 meV or a significant phase change. The unified QW peak positions imply that the STM visualization of the buried Al/Ge interface is not caused by a local reflective phase shift or a film thickness variation.^[^ ^13^, ^15^, ^16^ ^]^

In previous studies, the ZBCD was proved to be related to EPS,^[^ ^2^, ^22^, ^23^ ^]^ and the visualization of the buried interface may result from local electron diffuse scattering.^[^ ^14^ ^]^ Now, we analyze the ZBCD at different positions of the Moiré pattern and demonstrate that the STM‐visualized pattern on the Al/Ge film is not related to this mechanism. The EPS is characterized by the electron‐phonon spectral function (Eliashberg function) *α* ^2^ *F*(*ω*), which can be obtained from the d^2^I/dV^2^ spectra.^[^ ^2^, ^22^, ^24^ ^]^ The QW states have a significant influence on the strength of EPS,^[^ ^2^ ^]^ leading to modulations of the intensity in d^2^I/dV^2^ spectra (see Section S7, Supporting Information). Despite the influence from QW states, the lateral change in the local d^2^I/dV^2^ spectra is negligible. Figure 3f shows the d^2^I/dV^2^ spectra obtained by numerical differentiation of the dI/dV spectra in Figure 3e. It is evident that the peak position of 32 mV (matching the energy of phonons in Al^[^ ^25^, ^26^, ^27^ ^]^) as well as the peak values are both independent of the lateral position. This lateral homogeneity of the d^2^I/dV^2^ spectra indicates that there is no observable variation in the local EPS and thus, the STM observed pattern is not related to a local electron diffuse scattering at the interface.

The width of the QW peaks depends on the lateral position. For example, the dI/dV peaks measured at positions with lower apparent height (red curves in Figure 3a,e) exhibit sharper profiles than those taken at high apparent height positions (blue curves in Figure 3a,e). A similar phenomenon has also been observed in STS measurements of Pb/Si systems.^[^ ^28^, ^29^ ^]^ Since the properties of the barrier layers affect the coherence of these QW states, as previous work has demonstrated that interfacial interactions significantly modify the profile of QW peaks,^[^ ^29^ ^]^ the electronic states at the Al/Ge interface have a considerable influence on the STM results. Therefore, the modulation of the peak width is correlated with the spatial distribution of MIGS. To understand the lateral variation of the peak width, a 1D model is introduced to describe the electron transmission in an STM measurement. The Al film is treated as a Fabry‐Perot interferometer with two reflective interfaces, i.e., the Al/Ge interface and the vacuum/Al interface, as shown in Figure 2b. An electron can experience multiple reflections in the Al cavity, and the transmissivity of a coherent electron is a function of the electron energy of *E*. In an ideal interferometer (with no dissipation within the cavity), the transmissivity of a coherent electron is given by the Fabry‐Perot spectral function:^[^ ^22^, ^30^, ^31^ ^]^

(1) $$T=\frac{1}{1+\frac{4f^{2}}{\pi^{2}}\sin^{2}\left[k_{z}\left(E\right)L+\frac{\Phi }{2}\right]}\;$$

here, *f* is the interferometer finesse and is a function of the electron reflectivity at the two interfaces. In the Al cavity, *f* depends on the interfacial states. Typically, a higher local density of states results in a higher tunneling rate across the Al/Ge boundary, i.e., a lower reflectivity and a smaller *f*. *k*_z_(*E*) is the electron wavevector along the growth direction, and *L* is the thickness of the Al film. Φ is the sum of the reflective phase shift at the Al/Ge interface and the Al surface, and the value is independent of the lateral position (as studied in Figure 3e).

**Figure 4:**
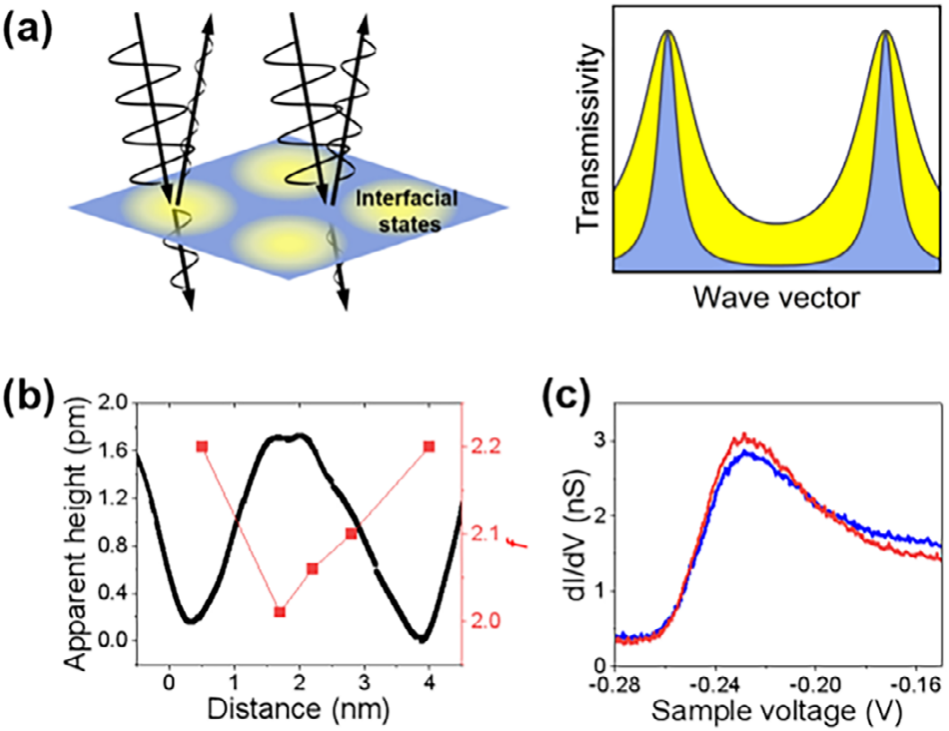
Lateral variation of the interfacial electron reflectivity. a) Schematic of lateral changes in electron transmissivity. Interfacial states induce a periodic lateral modulation of electron reflectivity, leading to variations in the transmissivity through the Al cavity. The transmissivity profiles at different lateral positions are filled in the corresponding colors. b) The STM apparent height and fitted values of *f* acquired along the white dashed line in Figure 3d. The *f* decreases with increasing apparent height. c) dI/dV spectra (*V*_s_ = −50 mV, *I*_t_ = −135 pA, *V*_mod_ = 3 mV) of the QW state at −227.5 meV measured at two lateral positions marked by the red and blue crosses in Figure 3d, respectively. The spectrum obtained at the position with lower apparent height (red) shows a sharper peak.

**Figure** **4**a schematically shows the influence of interfacial states on the electron transmissivity of *T*. The *T* exhibits an oscillatory behavior with increasing *k*_z_(*E*). The peak value of *T* appears when the electron wavevector matches the QW coherent condition of the Al film, which is given by

(2) $$2k_{z}\left(E_{n}\right)L+\Phi =2n\pi$$

where *n* is the quantum number. The wavevector difference between the neighboring QW states of *n*+1 and *n*, denoted as *∆k*_z_, can be calculated as *∆k*_z_ = π/*L*. We have *∆k*_z_ ≈0.03 Å^−1^ in an Al film of *L = *10 nm. Since the *∆k*_z_ is significantly smaller than the size of the Al Brillouin zone of 1.55 Å^−1^, the derivative of *k*_z_(*E*) to *E*, denotes as δ*k*_z_/δ*E*, is approximately to

(3) $$\frac{\delta k_{z}}{\delta E}\;{|}_{E_{n}}=\frac{\pi }{L\Delta E_{n}}\;$$

where *∆E_n_* is the energy difference between the QW states of *n* and *n*+1.

In realistic conditions of STM measurements on an Al/Ge film, the differential conductance can be written as a sum of two parts, the coherent tunneling part and the incoherent tunneling part, respectively. Bring Equation (3) to Equation (1), the differential conductance near the Fermi‐Level has a relation of the following:^[^ ^22^, ^31^ ^]^

(4) $$\begin{array}{ccc} \frac{dI}{dV} & \propto & A\left(E\right)\cdot \frac{1}{1+\frac{4f^{2}}{\pi^{2}}\sin^{2}\left[\frac{\pi }{\Delta E_{nF}}\left(E-E_{nF}\right)\right]}\\ & & +\;B\left(E\right)\;\mathrm{for}\;\left(E_{F}<E<E_{nF+1}\right) \end{array}$$

where the coefficient A(*E*) is a smooth function of *E* and is dependent on the tip properties and the tip setup with respect to the Al surface. B(*E*) is the incoherent tunneling term describing the contributions of electrons dissipated before reaching the Al/Ge interface and is also normally a smooth function of *E*. *E_F_* is the Fermi‐Level, *E_nF_* and *E_nF_*_+1_ are the energies of the first and second QW states above the Fermi‐Level, and *∆E_nF_* is the energy difference between the QW states just below and above the Fermi‐Level. Equation (4) indicates that a peak value of the differential conductance appears at the electron energy of *E_nF_*, corresponding to a QW state. The full width at half maximum (FWHM) of the peak is dominantly determined by the coherent tunneling term and is a function of *f*, expressed as *∆E*_FWHM_ ≈2Sin^−1^(π/2*f*) *∆E_nF_*/π. Therefore, a larger *f* corresponds to a sharper peak in the dI/dV spectra.

By fitting the dI/dV spectra to Equation (4), the values of *f* for each curve in Figure 3e are determined. It is worth noting that the data obtained at sample biases below + 35 mV are excluded from the fittings, because the EPS results in an “inelastic tunneling dip” in this bias range.^[^ ^22^, ^23^ ^]^ Considering that the dI/dV spectra shown in Figure 3e were taken in the same tip setup and the fitting to the data was performed in a small energy range of 35 <*E* <150 meV, A(*E*) was treated as a constant. The QW energy difference of *∆E_nF_* in a 10 nm thick Al film is 332 meV, which is obtained by measuring the peak positions in the dI/dV spectrum. Fitting results are shown in dashed lines in Figure 3e, which can qualitatively reproduce our experimental results. The fitted value of B(*E*) for each curve is at least one order of magnitude smaller than the coherent term (except the black colored curve obtained on a defected site), indicating that the coherent tunneling is dominant in STM measurements. Figure 4b shows the STM apparent heights and the fitted values of *f* obtained at different positions along the white dashed line in Figure 3d. We clearly see that a higher *f* (or a sharper dI/dV peak) corresponds to a lower apparent height. According to Equation (1), a higher *f* leads to a lower transmissivity of coherent electrons. The suppression of electron transmissivity would result in a lower local apparent height on the atomically flat Al surface where the tip approaches to the sample to sustain the current in constant‐current scanning mode. Our model is in good agreement with the *f*‐dependent apparent height. Figure 4c shows the dI/dV spectra of the QW state at −227.5 meV measured at two lateral positions marked by the red and blue crosses in Figure 3d. Here, it is seen that the width variation of the spectra peak is consistent with the observations in Figure 3e, i.e., a sharper peak corresponds to a measuring position with a lower STM apparent height. This result indicates that the position dependence of *f* is almost unchanged in the electron energy range of several hundreds meV. Therefore, the surface pattern in Figure 1c (taken at sample bias of *V*_s_ = −60 mV) and Figure 3d (*V*_s_ = −50 mV) reflects the spatial variation of *f* for electrons near the Fermi‐Level.

We argue that the lateral changes in the interferometer finesse *f* are caused by the variations of electron reflectivity at the Al/Ge interface. In the realistic vacuum/Al/Ge interferometer with dissipation, the *f* is given by^[^ ^30^, ^31^ ^]^

(5) $$f=\frac{\pi {\left(R_{1}R_{2}\right)}^{1/4}\cdot e^{-L/2\lambda }}{1-\sqrt{R_{1}R_{2}}\cdot e^{-L/\lambda }}\;$$

where *R*_1_ and *R*_2_ are the electron reflectivity on the Al/Ge interface and Al/vacuum interface, respectively. The *R*_2_ on the single crystalline Al(111) is considered as a constant. The λ is the electron mean free path, which describes the effects of diffuse scatterings from lattice defects or phonons. The presence of defects is related to an energy shift in the peaks in dI/dV spectra (black curve in Figure 3e). All the dI/dV spectra used for analyzing *f* were obtained from defect‐free regions, which have a constant dI/dV peak position of 104.5 mV. On the other hand, as shown in Figure 3f, the strength of EPS is independent of the lateral position. Therefore, the lateral change in λ is negligible as the scattering from defects and phonons is laterally homogeneous, and the variations of *f* are attributed to changes in *R*_1_. A parameter “relative reflectivity change”, denoted as *∆R*_1_/*R*_1_, is introduced to quantify the lateral variation in reflectivity. It is defined by the relation $\Delta R_{1}/\;R_{1}=2\left(R_{1}-{R_{1}}^{\prime }\right)/\left(R_{1}+{R_{1}}^{\prime }\right)$, where *R*_1_ and *R*_1_’ represent the reflectivity at two distinct positions. By substituting the fitted values of *f* into Equation (5), the reflectivity variation between the blue and red marked positions in Figure 3d is calculated to be *∆R*_1_/*R*_1_ = 22%. The complete calculation process is provided in Section S10 (Supporting Information).

**Figure 5:**
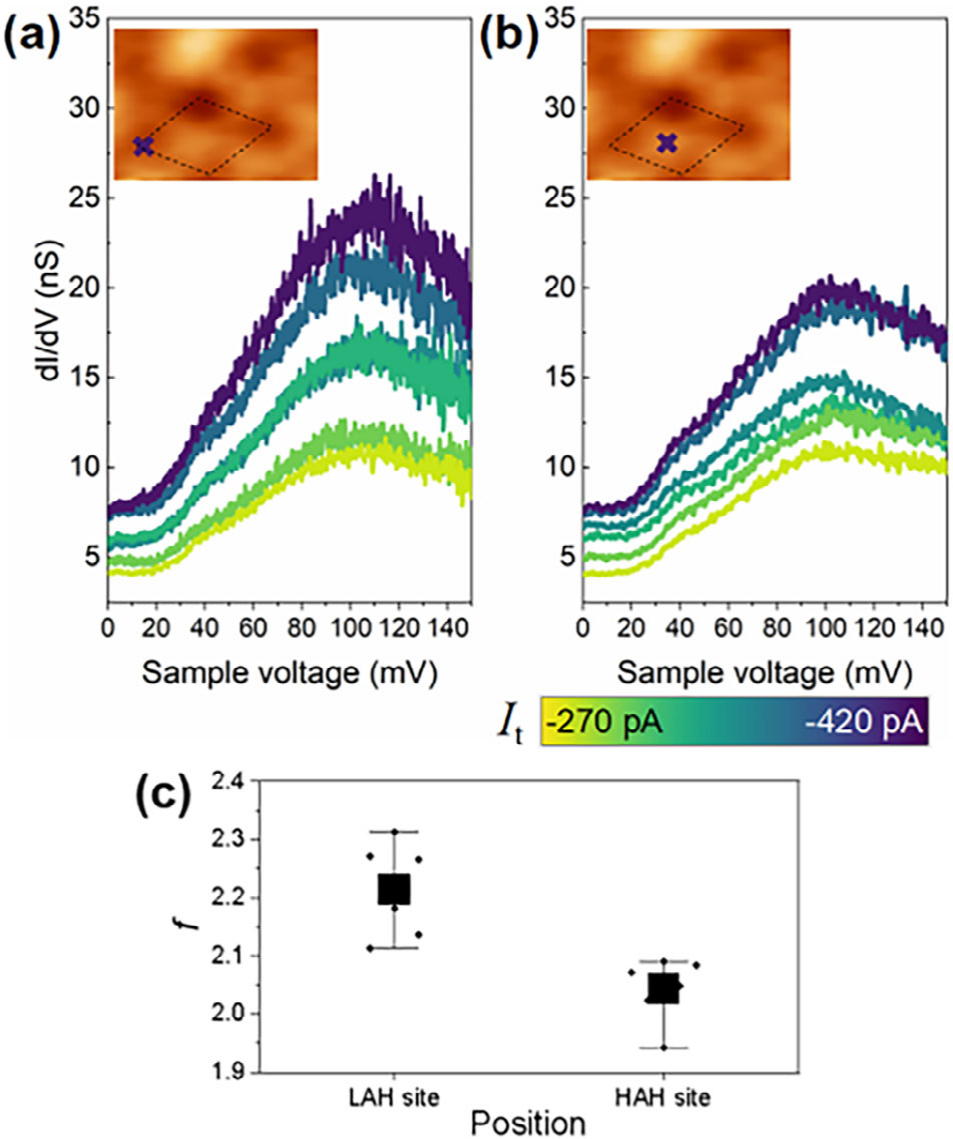
Local dI/dV spectra at different tip heights. a,b) Six groups of local dI/dV spectra obtained with various tip heights. The measurements are performed at *V*_s_ = −50 mV and *V*_mod_ = 2 mV. The tip heights are tuned by adjusting tunneling current *I*_t_, which ranges from −270 to −420 pA. Insets: measurement positions. c) Fitted *f* values from (a,b). The low‐apparent‐height (LAH) site yields a larger *f* than the high‐apparent‐height (HAH) site.

To reinforce the validity of the variation in *R*_1_, STS measurements with different tip settings have been studied. By varying the tunneling current, the tip height, and *R*_2_ are changed. This adjustment leads to significant variations in the profile of the spectral curves, consequently affecting the fitted values of *f*. However, the extracted value of *∆R*_1_/*R*_1_ should be unchanged, as the interface states involved in coherent tunneling remain unchanged. **Figure** **5**a,b shows six groups of dI/dV results obtained with shorter tunnel junctions, with measurement positions presented in the insets. The tip heights in these measurements are tuned by adjusting the tunneling current from −270 to −420 pA. The fitted values of *f* are shown in Figure 5c, demonstrating that the position with lower apparent height yields larger *f*. The averaged *∆R*_1_/*R*_1_ calculated from Figure 5c is 19%, comparable to the result obtained in Figure 3e.

The local dI/dV spectra on samples with various thicknesses are studied (see Section S8, Supporting Information). The position dependence of the QW peaks in these experiments is all qualitatively consistent with Figure 4a, indicating that the physical model matches the experimental results of various thicknesses. Spatial dI/dV maps of various sample biases are studied, the details are shown in the supporting information (see Section S9, Supporting Information). The contrast of the surface pattern in the dI/dV maps changes at different sample biases. The lowest contrast appears at the bias corresponding to the QW energy of *E_n_*, indicating that the spatial variation of differential conductance is suppressed when the electron is perfectly matched to the QW coherent condition. This result is also in good agreement with our physical model of Equation (4).

## 3 Conclusion

In conclusion, we have shown that the interfacial atomic structures in an Al/Ge epitaxial film can be observed by STM imaging. Different from the previously studied metal/semiconductor thin films, the electronic scattering at the atomically flat Al/Ge interface exhibits lateral uniformity in both the reflective phase and electron‐phonon scattering strength. We have introduced a physical model based on a Fabry‐Perot interferometer to describe the electron transmission in the STM measurements, which successfully explains the experimental observations. From this model, we conclude that the visualization of the buried interface results from the lateral change in interfacial electron reflectivity induced by interfacial states. The lateral modulations of electron reflectivity may be general in various hetero‐interfaces. For heterostructures with superior crystalline quality and thickness within the electron coherence length, where coherent electron tunneling occurs with limited dissipation, our methodology enables the probing of interfacial state‐mediated tunneling effects at atomically flat hetero‐interfaces. We believe that our observations have potential applications for non‐destructive detection in the van der Waals interface of layered materials or incoherent heterostructures with exotic interlayer couplings.

## Conflict of Interest

The authors declare no conflict of interest.

## Supporting information



Supporting Information

## Data Availability

The data that support the findings of this study are available from the corresponding author upon reasonable request.

## References

[1] Y. F. Zhang , J. F. Jia , T. Z. Han , Z. Tang , Q. T. Shen , Y. Guo , Z. Q. Qiu , Q. K. Xue , Phys. Rev. Lett. 2005, 95, 096802. 16197236 10.1103/PhysRevLett.95.096802

[2] M. Schackert , T. Märkl , J. Jandke , M. Hölzer , S. Ostanin , E. K. U. Gross , A. Ernst , W. Wulfhekel , Phys. Rev. Lett. 2015, 114, 047002. 25679904 10.1103/PhysRevLett.114.047002

[3] Y. Guo , Y. F. Zhang , X. Y. Bao , T. Z. Han , Z. Tang , L. X. Zhang , W. G. Zhu , E. G. Wang , Q. Niu , Z. Q. Qiu , J. F. Jia , Z. X. Zhao , Q. K. Xue , Science 2004, 306, 1915. 15591197 10.1126/science.1105130

[4] W. M. J. van Weerdenburg , A. Kamlapure , E. H. Fyhn , X. Huang , N. P. E. van Mullekom , M. Steinbrecher , P. Krogstrup , J. Linder , A. A. Khajetoorians , Sci. Adv. 2023, 9, adf5500. 10.1126/sciadv.adf5500 PMC9977180 36857452

[5] T. Zhang , P. Cheng , W. J. Li , Y. J. Sun , G. Wang , X. G. Zhu , K. He , L. Wang , X. Ma , X. Chen , Y. Wang , Y. Liu , H. Q. Lin , J. F. Jia , Q. K. Xue , Nat. Phys. 2010, 6, 104.

[6] I. B. Altfeder , X. Liang , T. Yamada , D. M. Chen , V. Narayanamurti , Phys. Rev. Lett. 2004, 92, 226404. 15245244 10.1103/PhysRevLett.92.226404

[7] F. Vigneau , R. Mizokuchi , D. C. Zanuz , X. Huang , S. Tan , R. Maurand , S. Frolov , A. Sammak , G. Scappucci , F. Lefloch , S. De Franceschi , Nano. Lett. 2019, 19, 1023. 30633528 10.1021/acs.nanolett.8b04275

[8] M. Valentini , O. Sagi , L. Baghumyan , T. de Gijsel , J. Jung , S. Calcaterra , A. Ballabio , J. A. Servin , K. Aggarwal , M. Janik , T. Adletzberger , R. S. Souto , M. Leijnse , J. Danon , C. Schrade , E. Bakkers , D. Chrastina , G. Isella , G. Katsaros , Nat. Commun. 2024, 15, 169. 38167818 10.1038/s41467-023-44114-0 PMC10762135

[9] O. Sagi , A. Crippa , M. Valentini , M. Janik , L. Baghumyan , G. Fabris , L. Kapoor , F. Hassani , J. Fink , S. Calcaterra , D. Chrastina , G. Isella , G. Katsaros , Nat. Commun. 2024, 15, 6400. 39080279 10.1038/s41467-024-50763-6 PMC11289319

[10] I. B. Altfeder , D. M. Chen , K. A. Matveev , Phys. Rev. Lett. 1998, 80, 4895.

[11] Y. Jiang , K. H. Wu , Z. Tang , P. Ebert , E. G. Wang , Phys. Rev. B 2007, 76, 035409.

[12] M. L. Tao , H. F. Xiao , K. Sun , Y. B. Tu , H. K. Yuan , Z. H. Xiong , J. Z. Wang , Q. K. Xue , Phys. Rev. B 2017, 96, 125410.

[13] H. Kim , Y. Hasegawa , Phys. Rev. B 2016, 93, 075409.

[14] I. B. Altfeder , K. A. Matveev , A. A. Voevodin , Phys. Rev. Lett. 2012, 109, 166402. 23215098 10.1103/PhysRevLett.109.166402

[15] I. B. Altfeder , V. Narayanamurti , D. M. Chen , Phys. Rev. Lett. 2002, 88, 206801. 12005588 10.1103/PhysRevLett.88.206801

[16] W. B. Jian , W. B. Su , C. S. Chang , T. T. Tsong , Phys. Rev. Lett. 2003, 90, 196603. 12785967 10.1103/PhysRevLett.90.196603

[17] Y. Jiang , J. D. Guo , P.h. Ebert , E. G. Wang , K. H. Wu , Phys. Rev. B 2010, 81, 033405.

[18] T. Nishimura , K. Kita , A. Toriumi , Appl. Phys. Lett. 2007, 91, 123123.

[19] A. Dimoulas , P. Tsipas , A. Sotiropoulos , E. K. Evangelou , Appl. Phys. Lett. 2006, 89, 252110.

[20] S. Ciraci , A. Baratoff , I. P. Batra , Phys. Rev. B 1991, 43, 7046. 10.1103/physrevb.43.7046 9998168

[21] V. Heine , Phys. Rev. 1965, 138, A1689.

[22] K. Wang , X. Zhang , M. M. T. Loy , T. C. Chiang , X. Xiao , Phys. Rev. Lett. 2009, 102, 076801. 19257703 10.1103/PhysRevLett.102.076801

[23] X. Wu , C. Xu , K. Wang , X. Xiao , Phys. Rev. B 2015, 92, 035434.

[24] E. Minamitani , N. Takagi , R. Arafune , T. Frederiksen , T. Komeda , H. Ueba , S. Watanabe , Prog. Surf. Sci. 2018, 93, 131.

[25] S. Y. Savrasov , D. Y. Savrasov , O. K. Andersen , Phys. Rev. Lett. 1994, 72, 372. 10056414 10.1103/PhysRevLett.72.372

[26] G. G. Rusina , S. V. Eremeev , S. D. Borisova , I. Y. Sklyadneva , E. V. Chulkov , Phys. Rev. B 2005, 71, 245401.

[27] R. Bauer , A. Schmid , P. Pavone , D. Strauch , Phys. Rev. B 1998, 57, 11276.

[28] S. M. Lu , M. C. Yang , W. B. Su , C. L. Jiang , T. Hsu , C. S. Chang , T. T. Tsong , Phys. Rev. B 2007, 75, 113402.

[29] Y. S. Fu , S. H. Ji , T. Zhang , X. Chen , J. F. Jia , Q. K. Xue , X. C. Ma , Chin. Phys. Lett. 2010, 27, 066804.

[30] M. Fox , Quantum Optics: An Introduction, Vol. 15, Oxford University Press, Oxford UK 2006.

[31] J. J. Paggel , T. Miller , T. C. Chiang , Science 1999, 283, 1709. 10073930 10.1126/science.283.5408.1709

