# Supplementary material for: Atomically Resolved Electron Reflectivity at a Metal/Semiconductor Interface
Source: Adv Sci (Weinh). 2025 Nov 29;13(9):e15182. doi: 10.1002/advs.202515182 (PMC12903964; doi:10.1002/advs.202515182)
Supplement: Supplementary file 1 — Supporting Information [file ADVS-13-e15182-s001.docx]

**SUPPORTING INFORMATION**

**Atomically resolved electron reflectivity at a metal/semiconductor interface**

Ding-Ming Huang,^1,2,3,*^ Jian-Huan Wang,^1,2,3^ Jie-Yin Zhang,^2^ Yuan Yao,^2^ Hongqi Xu,^1,3,†^ and Jian-Jun Zhang^2,4,‡^

*^1^Beijing Academy of Quantum Information Sciences, Beijing 100193, China*

^2^*Beijing National Laboratory for Condensed Matter Physics and Institute of Physics,*

*Chinese Academy of Sciences, Beijing 100190, China*

*^3^Beijing Key Laboratory of Quantum Devices and School of Electronics, Peking University, Beijing 100871, China*

*^4^Hefei National Laboratory, Hefei 230088, China*

* [huangdm@baqis.ac.cn](mailto:huangdm@baqis.ac.cn);† [hqxu@pku.edu.cn](mailto:hqxu@pku.edu.cn); ‡ jjzhang@iphy.ac.cn

（Dated: Aug 8, 2025）

**SUPPORTING INFORMATION Section S1：**

**A simulation of Moiré pattern based on the interfacial lattice.**

In Fig. SI 1a, on the left part we schematically plot the possible lattice configuration of the Al/Ge interface, and on the right part the corresponding simulated Moiré pattern is shown. The unit cell is marked by dashed diamond. This simulation is generated by first filtering the q_3_ and q_4_ points from Fig. 1h of the main text and then applying an inverse FFT, yielding qualitative agreement with the measurements. A slight deviation from the measurement occurs in the simulated pattern, which may result from oversimplified treatment of both the interfacial coupling, lateral deviation of the interfacial lattice and the tip setup. It is necessary to point out that this simulation provides only a qualitative result. Since the Moiré pattern is correlated to the interfacial states, in-depth considerations of interfacial coupling will be necessary for more accurate simulations.

**Fig. SI 1.** Schematic of a possible interfacial lattice and the corresponding Moiré pattern. Unit cells are marked by dashed diamonds.

**SUPPORTING INFORMATION Section S2：**

**The correlation between the Moiré** **pattern and the electronic coherent states**

Decoherence of electron can be achieved by increasing the temperature, as the electron mean free path λ decreases due to the enhancement of electron-phonon scattering (EPS). Fig. SI 2a and 2c show the FFT images of STM morphology on a 25 nm thick Al/Ge film obtained at 10 K and room temperature (RT), respectively. Notably, The measurements were performed using the same STM system and tip, but at different locations on the sample due to unavoidable thermal drift. Despite this positional variation, the features in Fig. SI 2 remain reproducible and representative, which we attribute to the homogeneous single-crystalline epitaxial film. The reciprocal points of the Al/Ge interfacial lattice are shown at 10 K and vanish at RT. The dI/dV spectra obtained at 10K and RT are shown in Fig. SI 2b and 2d, respectively. The differential conductance is a smooth function of sample bias at RT, as the decoherence of electrons causes the vanishing of the QW peaks. The presence of the Moiré pattern coincides with the presence of QW states, further indicating that the Moiré pattern is caused by the coherent electronic states.


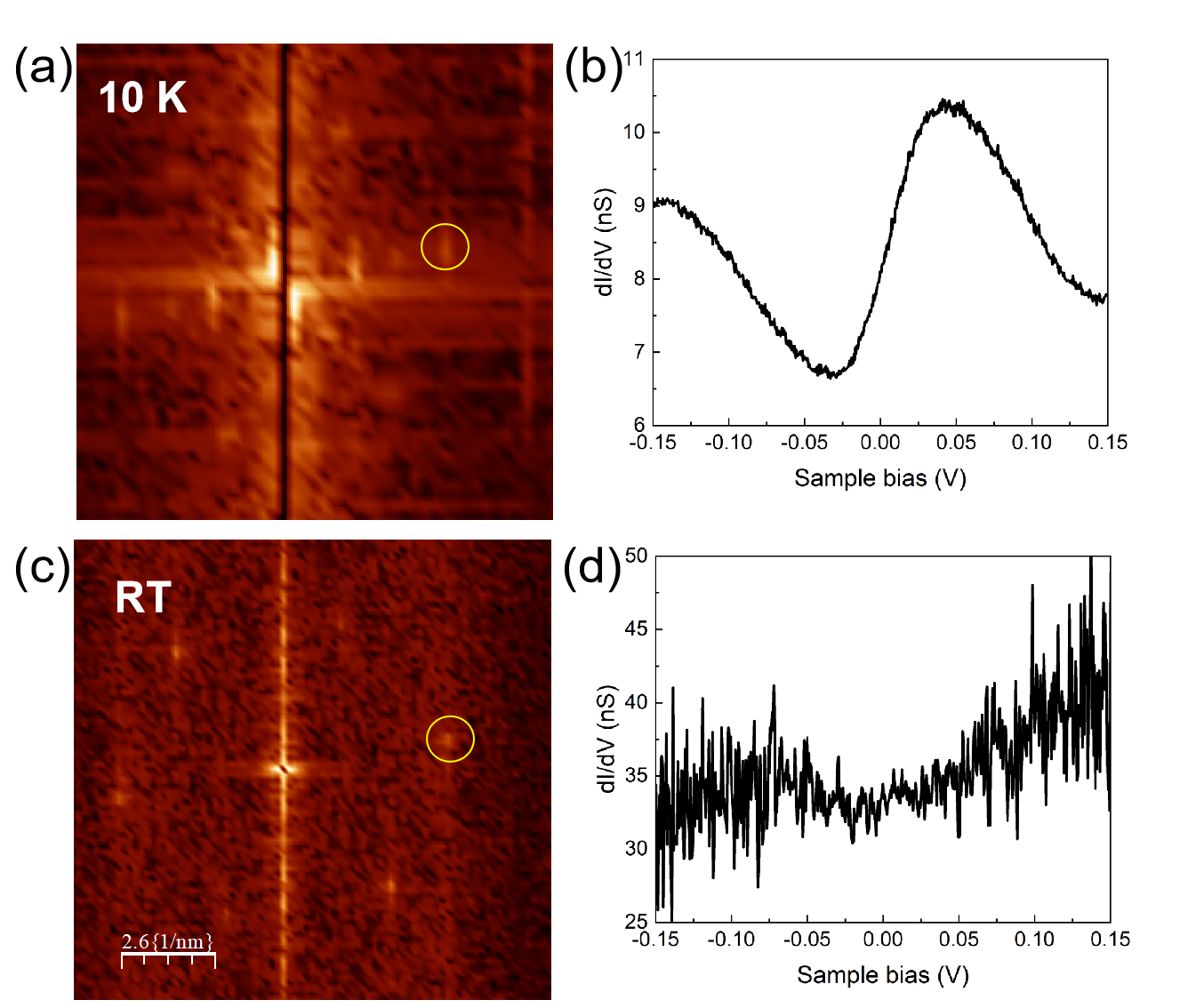


**Fig. SI 2.** Decoherence of reflective electron with increasing of temperature in a 25 nm thick Al/Ge film. (a) FFT image of the surface morphology at 10 K. A reciprocal point of Al lattice is marked by yellow circle. (b) dI/dV spectrum on surface at 10 K (-50 mV, -345 pA and 28 mV modulation). (c) FFT image of the surface morphology at RT. (d) dI/dV spectrum on surface at RT (-50 mV, -1.14 nA and 5 mV modulation). A reciprocal point of Al lattice is marked by yellow circle.

**SUPPORTING INFORMATION Section S3：**

**Characterizations of the distortion of Al lattice**

The surface lattice of a 10 nm thick Al/Ge film is characterized by an in-situ Low-Energy Electron Diffraction (LEED). Figure SI 3a and Fig. SI 3b are LEED images taken at RT and 35 K, respectively. To achieve the highest imaging contrast, the energy of induced electrons is set at 132 eV, and only 3 reciprocal points of the Al surface lattice are visualized. The contrast of LEED images enhances with the decreasing of temperature, but the LEED pattern is invariant. The absence of Moiré points indicates that the reconstruction of the Al surface lattice is negligible (at least smaller than the resolution of LEED), and the Moiré pattern in STM measurements is dominant by local electronic property. The STM image on a stacking fault is shown in Fig. SI 3c, where the disorder morphology is only visible within 3 nm from the stacking fault. The Moiré pattern near the stacking fault (beyond 3 nm) remains the same as the pattern on defect-free region. While, the stacking fault induced distortion at least influence the lattice within lateral 15 nm, which can be observed by the height variations in the STM image (Fig. SI 3d and 3e). Thus, the Moiré pattern is independent of lattice distortion.

In summary, the Moiré pattern in Al/Ge film is independent of lattice reconstruction and distortion.

**Fig. SI 3.** Surface lattice and defect induced distortion on a 10 nm thick Al/Ge film. (a) and (b) LEED images at RT and 35 K, respectively. (c) Moiré pattern near a stacking fault. (d) STM morphology on a stacking fault. (e) Profile of height taken from the gray line in (d).

**SUPPORTING INFORMATION Section S4:**

**Pre-treatment of STM tip for dI/dV studies.**

Achieving atomic resolution image on low Miller index metal surfaces (such as Al(111)) is always challenging due to the “plain” surface DOS and small lattice constant, especially for an s-wave STM tip with “plain” DOS at Fermi-level (Phys. Rev. Lett. **65**, 448 (1990)). The atomic resolution image in **Fig. 1b of the main text** is obtained by a “Ge decorated tip”, which is obtained by picking up a Ge cluster (or a Ge atom). However, this tip cannot be utilized in STS measurements due to the unpredictable DOS of the attached cluster. In order to acquire STS spectra with high energy precision, tips for STS measurements are pre-treated on an Au(111) substrate until a standard dI/dV spectrum of Au surface is achieved (an example is shown in Fig. SI 4a). The calibrated tips present an s-wave feature and typically have a reduced spatial resolution.

To elucidate that the suppressed resolution in **Fig. 3d** results from the tip configuration, we have also imaged the sample with a relatively sharper tip. Figure SI 4b shows the STM image, and 4c presents a height profile. The observed Moiré pattern matches that in **Fig. 1c**.

**Fig. SI 4.** (a) dI/dV spectrum taken on Au(111) surface after the calibration of tip’s DOS. (b) STM image of the 10 nm Al/Ge film acquired with a sharp tip (*V_s_* = -150 mV, *I_t_* = -135 pA). (c) Height profile across the Moiré pattern.

**SUPPORTING INFORMATION Section S5:**

**Analysis of the peak positions in dI/dV spectra.**

The peak positions of the quantum well state across dI/dV spectra were determined by fitting the coefficient *E_nf_* in Eq. (4). The fitted peak values (corresponding to E_nf_) for the curves in **Fig. 3e** are shown in Fig. SI 5. The deviation of the peak values in the superlattice equals ± 3.5 mV, nearly an order of magnitude smaller than the peak shift induced by a point defect in the lattice (Δ = -25.5 mV for the black-marked site). The systematic measurement error may cause this slight deviations, due to the lock-in amplitude of 5 mV. Despite this systematic error, a subtle increasing trend in peak values is observed with decreasing distance to the point defect. We speculate that this trend is likely related to a point defect induced perturbation, similar to the screening effect observed within a lateral range of ~1 nm around the defect [15]. Even if this deviation is interpreted as a reflective phase shift of ~2π/100, the value remains significantly smaller than those reported in comparable material systems [12]. Therefore, the Al/Ge interface can be identified as phase-uniform in reflectivity, and these minor peak shifts have no impact on the paper’s conclusions.

Fig. SI 5. Quantum well state energies *E_nf_* extracted from STS peaks.

**SUPPORTING INFORMATION Section S6:**

**Point defects in the Al/Ge films.**

We speculate that the defect site (black marked) in **Fig. 3d** is caused by Ge dopant. Below is a detailed discussion of our assumption.

Figure SI 6a shows an atomic resolution STM image of 3 types of point defects and the corresponding height profiles, from top to bottom are “bright spot”, surface vacancy and adatom, respectively. The apparent height of “black spot” defects in **Fig. 1c** are the same as that of vacancies. Therefore, we can determine that the “dark spots” in **Fig. 1c** are vacancies. Figure SI 6b shows the height profile of the point defect in **Fig. 3d**, and the apparent height is close to that of “bright spot” in Fig. SI 6a, indicating that these defects are the same. According to the atomic resolution image of “bright spot” in Fig. SI 6a, where the most top atoms are clearly visible, we can determine that this defect is located under the Al surface. Figure SI 6c to f schematically show 4 types of point defects that may form under the Al surface, which are interfacial Ge vacancy, interfacial Al dopant, Al vacancy and Ge dopant, respectively. The dI/dV spectrum on the “bright spot” defect is shown in **Fig. 3e**, where the QW peak shift of black curve (towards negative bias) indicates a higher effective thickness or an increase in effective energy barrier (PRB **81**, 033405 (2010)). We speculate that the Ge dopant (Fig. SI 6f) is the only reason that could lead to the STM results we obtained.

The interfacial Ge vacancy (Fig. SI 6c) may cause a decreased apparent height at surface due to the strain induced by the missing atom. This defect breaks three Ge-Ge valence bonds, and the dangling bonds may attract an Al atom, forming an Al dopant (Fig. SI 6d). The interfacial Al dopant result in a slight increase in effective thickness. However, similar to Ga dopant on Si substrate (PRB **81**, 033405 (2010)), an Al dopant on Ge may also induce a screening of the interfacial barrier, leading an additional QW peak shift towards positive bias. Al vacancy (Fig. SI 6e) may result in a slight decrease of the effective thickness, leading a QW peak shift to positive bias and a decrease in apparent height. Different to the 3 types of defects being mentioned above, Ge dopants (Fig. SI 6f) may lead to STM results we obtained. The relatively larger size of Ge atoms may result in an increased apparent height and a corresponding QW shift. Furthermore, the fitting of dI/dV (black curve in **Fig. 3e**) demonstrate a significant incoherent tunneling at the defect site. The fitting value of B(E) in Eq. (4) is comparable to the value of A(E) (A/B = 1.1). This result indicates an enhanced incoherent scattering, which attribute to scatterings from the Ge dopant.

**Fig. SI 6.** Point defects in the material. (a) Atomic resolution STM image of point defects and the corresponding height profile. From top to bottom are “bright spot”, surface vacancy and adatom, respectively. (b) The STM image of **Fig. 3d** in manuscript and the height profile. The apparent height of +5 pm proves that the defect is not a surface adatom. (c) to (f) Schematic of 4 types of point defects that can occur in Al films. (c) interfacial Ge vacancy, (d) interfacial Al dopant, (e) bulk Al vacancy and (f) Ge dopant, respectively. The red curves schematically illustrate the lateral variation of effective thickness caused by corresponding defects.

**SUPPORTING INFORMATION Section S7:**

**Details of local d^2^I/dV^2^ spectra.**

The QW states significantly influence the electron-phonon-interaction (EPI) in our Al/Ge films, leading to modulations of intensity in d^2^I/dV^2^ spectra, as also reported in Phys. Rev. Lett. **114**, 047002 (2015). In our experiments, the d^2^I/dV^2^ peak positions at positive and negative biases are perfectly matched, but the intensity varies. Despite the asymmetric feature of d^2^I/dV^2^ spectra, unchanged d^2^I/dV^2^ peaks at different positions indicate that the Moiré pattern is independent of EPI. Below are the details.

Local d^2^I/dV^2^ spectra on a 10 nm thick Al/Ge sample, measured with bias modulation of 2 mV, are shown in Fig. SI 7a, red and blue curves are measured on positions corresponding to colored marks in Fig. 3d. The peaks at ± 32 mV and ± 23 mV (triangle marked) result from the EPI in Al [25,26]. The peak value at positive bias is approximately double of that at negative bias, this asymmetry persists across various measurements and independent of bias modulation or tunneling current. We attribute this asymmetry to the result of QW-states-modulated-electron-phonon-interaction (PRL **114**, 047002 (2015)). The corresponding local dI/dV spectra are shown in Fig. SI 7b (displayed in corresponding colors), where the QW peak is close to the Fermi-level and results in a steep slope at positive bias, supporting our hypothesis. In order to reduce the influence from QW states, we studied the d^2^I/dV^2^ spectra on a 3 nm Al/Ge film, where the Fermi-level is approximately centered between two QW peaks. Fig. SI 7c shows the d^2^I/dV^2^ data, and the measuring positions are marked in Fig. SI 7d with corresponding colors. The inset of Fig. SI 7d shows the dI/dV spectrum taken on this surface, which presents QW peaks at -380 mV and +440 mV, respectively. Fig. SI 7c shows a slight enhancement of d^2^I/dV^2^ at negative bias. This asymmetry is still attribute to the QW enhancement, as the QW peak at negative bias is slightly closer to the Fermi-level. These d^2^I/dV^2^ results demonstrate that the asymmetry is influenced by the positions of the QW peaks

Despite the asymmetric feature, the difference between d^2^I/dV^2^ spectra in Fig. SI 7a is negligible, indicating homogeneous EPI across different positions. Furthermore, we would like to point out that **if we have missed any of the ultra-fine EPI features, this EPI cannot result in a strong Moiré pattern.** In summary, the Moiré pattern is independent of EPI.

**Fig. SI 7.** Local d^2^I/dV^2^ spectra on the Moiré pattern. (a) and (b) Local d^2^I/dV^2^ spectra and dI/dV spectra on a 10 nm Al/Ge film (*V*_s_ = -50 mV, *I*_t_ = -345 pA, and *V*_mod_ = 2 mV). These spectra are measured at the corresponding positions described in **Fig. 3d**. Triangles in (a) mark the d^2^I/dV^2^ peaks at ± 32 mV and ± 23 mV, resulting from electron-phonon interactions in Al. (c) Local d^2^I/dV^2^ spectra obtained on a 3 nm film (*V*_s_ = 25 mV, *I*_t_ = 21 pA, and *V*_mod_ = 3 mV). The triangles mark the voltage value of ± 32 mV. (d) STM image of the Moiré pattern on a 3 nm Al/Ge film. Inset shows the dI/dV spectrum taken on the surface (*V*_s_ = 25 mV, *I*_t_ = 41 pA, and *V*_mod_ = 3 mV). The colored crosses mark the measuring positions of the d^2^I/dV^2^ curves in (c).

**SUPPORTING INFORMATION Section S8:**

**dI/dV results on various film thicknesses.**

The dI/dV spectra on Al/Ge films show dramatic changes with increase of thicknesses, which result from the QW peak shift. This thickness-dependence is unique to QW states and distinguishes them from other surface or interface electronic states. An example of this QW shift is shown in Fig. SI 8a, which is obtained on a 10 nm thick Al/Ge sample. The thickness dependence of the dI/dV spectra on Al/Ge samples was studied and shown in Fig. SI 8c to 8h. Owing to the two-dimensional growth of the Al film, we can precisely control the thickness by modifying the deposition of Al. It is worth noting that the changes in STM resolution in Fig. SI 8d and 8e are due to the random tip shapes after pre-treatments (details can be found in **s**upporting information 4). Fig. SI 8c shows the dI/dV results presented in Fig. 3a of the paper main text. The dI/dV measuring positions are marked by crosses with corresponding colors, and the red site presents a lower apparent height than the blue one. A unit cell of the Moiré pattern is marked by a dashed diamond. The red curve presents lower dI/dV values near the Fermi-level than the blue one, i. e. a sharper QW peak. These dI/dV spectra agree with the physical model of Eq. (1). Fig. SI 8d to 8h show the STM results obtained on 3 nm, 4 nm, 6 nm, 7 nm and 8 nm samples, respectively. In order to minimize the error from thermal drift, the dI/dV spectra of these thicknesses (including the results in Fig. 3e of the paper main text) are obtained under “constant current mode”, where the tip height is readjusted by the feedback current before each individual measurement. A schematic of the “constant current mode” measurement is shown in Fig. SI 8b. The tip approaches the sample at positions with lower electron transmissivity, keeping the dI/dV values near Fermi-level constant and result in a higher QW peak. All the dI/dV data from Fig. SI 8d to 8h show higher QW peaks at low apparent height sites (red). This consistency across various samples indicates that the phenomenon results from interfacial properties and is independent of film thickness. Therefore, the Fabry-Perot interferometer model matches the experimental results under various thicknesses.

**Fig. SI 8.** Local dI/dV spectra on Al/Ge films with various thicknesses. (a) dI/dV spectra obtained from neighbored atomic steps on a 10 nm Al film. QW peaks present an energy shift of about -110 meV with the increasing of a single atomic step. Inset: STM image shows the atomic step. The dI/dV measurement sites are marked by colored crosses. (b) Schematic of dI/dV measurements under “constant current mode”. When STM tip approaches the sample positions with lower electron transmissivity, it results in a higher peak value. (c) Local dI/dV spectra and STM image obtained on a 2 nm thick Al/Ge film, which are the same data presented in Fig. 3a. (d) to (h) Local dI/dV spectra and STM images obtained on 3 nm, 4 nm, 6 nm, 7 nm and 8 nm thick Al/Ge samples, respectively. These dI/dV spectra are measured under “constant current mode”. The dI/dV measurement sites and the unit cells of Moiré pattern in STM images from (c) to (h) are marked by colored crosses and dashed diamonds, respectively. Blue dI/dV curves from (c) to (h) are obtained at positions (blue marks) with a higher apparent height. STS measurement parameters: (a) *V*_s_ = -450 mV, *I*_t_ = -125 pA, and *V*_mod_ = 25 mV; (c) *V*_s_ = -50 mV, *I*_t_ = -436 pA, and *V*_mod_ = 6 mV; (d) *V*_s_ = 21 mV, *I*_t_ = 115 pA, and *V*_mod_ = 6 mV; (e) *V*_s_ = 300 mV, *I*_t_ = 320 pA, and *V*_mod_ = 10 mV; (f) *V*_s_ = 21 mV, *I*_t_ = 51 pA, and *V*_mod_ = 5 mV; (g) *V*_s_ = 92 mV, *I*_t_ = 68 pA, and *V*_mod_ = 26 mV; (h) *V*_s_ = 55 mV, *I*_t_ = 251 pA, and *V*_mod_ = 4 mV.

**SUPPORTING INFORMATION Section S9:**

**Studies of spatial dI/dV maps.**

We utilize the self-correlation and FFT methods to study the Moiré period in dI/dV maps, these are the common methods applied in analysis of in-plane periods (PRB **89**, 235115 (2014) ).

Figure SI 9a to 9c present the dI/dV maps at sample biases of +203 mV, +102 mV and +82 mV, respectively. The corresponding self-correlation and FFT images of the dI/dV maps are shown in the left and right insets, respectively. The dI/dV spectrum taken at the mapping region is shown in Fig. SI 9d, and a QW peak sits at +79±3 mV. The peak position is different to the QW state in Fig. 3c of the paper main text, which may result from a slight thickness deviation between the measured samples. Cross-sectional profiles along the marked lines in self-correlation images are shown in Fig. SI 9e. The periodic oscillation of intensity is suppressed when the sample bias reduces to +82 mV, indicating a decrease in periodic correlation. Cross-sectional profiles along the marked lines in FFT images are shown in Fig. SI 9f. The intensity peaks at 3.6 nm^-1^ correspond to the reciprocal point of q_4_ in **Fig. 1g**. The peak value reduces with decreasing of sample bias from +203 mV to +82 mV, indicating a suppression in periodic discrepancy of dI/dV. According to the physical model of Eq. (1) in the paper’s main text, the lateral variation of the electronic transmissivity disappears when the electron wavevector perfectly matches to the coherent condition in a Fabry-Perot cavity. Therefore, the suppression of the Moiré pattern at the bias of a QW state is in good agreement with our physical model.

**Fig. SI 9.** dI/dV maps on a 10 nm Al/Ge film. (a), (b) and (c) dI/dV maps taken at bias of +203 mV, +102 mV and +82 mV, respectively. Corresponding self-correlation images and the FFT images are shown in the left and right insets, respectively. (d) dI/dV spectrum taken at the mapping region (*V*_s_ = 132 mV, *I*_t_ = 90 pA, and *V*_mod_ = 10 mV). A QW peak sits at bias of +79±3 mV. (e) and (f) Intensity Profiles along the solid lines in self-correlation images and FFT images, respectively. These profiles are shown in the corresponding colors of the solid lines.

**SUPPORTING INFORMATION Section S10：**

**Calculations of relative interfacial reflectivity *ΔR_1_/R_1_*.**

According to Eq. (5):

$f=\frac{\pi{(R_{1}R_{2})}^{1/4}{\cdot e}^{-L/2\lambda}}{1-\sqrt{R_{1}R_{2}}{\cdot e}^{-L/\lambda}}$, (5)

*f* can be written as:

$f=\frac{\pi{(R_{1})}^{1/4}\cdot K}{1-{{[(R_{1})}^{1/4}\cdot K]}^{2}}$. (6)

Here, the coefficient *K* is defined as $K={(R_{2})}^{1/4}{\cdot e}^{-L/2\lambda}$ and can be treated as a constant across measurements, as explained in the discussion of Equation (5) in the paper main text. Equation (6) is a quadratic equation with respect to the unknown variable of ${(R_{1})}^{1/4}\cdot K$. The analytical solution of this equation yields:

$R_{1}\cdot K^{4}=({\frac{\sqrt{{(\pi}^{2}/f^{2})+4}-\pi/f}{2})}^{4}$. (7)

By substituting the fitted values of *f* from the dI/dV spectra into Eq. (7), the values of $R_{1}\cdot K^{4}$ can be obtained. The uncertainty in *K* precludes an exact calculation of *R*_1_. However, the relative change of interfacial reflectivity *ΔR_1_/R_1_* can be derived by the two values of *f* obtained at distinct positions. Because,

$\Delta R_{1}/R_{1}=\frac{2(R_{1}-R_{1}’)}{R_{1}+R_{1}’}$=$\frac{2(R_{1}\cdot K^{4}-R_{1}’\cdot K^{4})}{R_{1}\cdot K^{4}+R_{1}’\cdot K^{4}}$， (8)

and the right side of the equation is simply a function of the two measured values of *f*.
